# Case Report: From multimodality imaging to catheter ablation of ventricular arrhythmias in arrhythmogenic mitral valve prolapse

**DOI:** 10.3389/fphys.2025.1654085

**Published:** 2025-08-22

**Authors:** Ali Alzammam, Faisal Alanazi, Sultan Alenazy, Abdulmahsen Alsalman, Abdulrahman Albadi, Maysan Almegbel, Ahmed Aljizeeri, Muneera Altaweel, Abdulmohsen Almusaad

**Affiliations:** ^1^ Department of Adult Cardiology, King Abdulaziz Cardiac Center, MNGHA, King Abdulaziz Medical City, Riyadh, Saudi Arabia; ^2^ Department of Adult Echocardiography, King Abdulaziz Cardiac Center, MNGHA, King Abdulaziz Medical City, Riyadh, Saudi Arabia; ^3^ Department of Advanced Cardiac Imaging, King Abdulaziz Cardiac Center, King Abdullah International Medical Research Center (KAIMRC), Ministry of National Guard Health Affairs (MNGHA), King Abdulaziz Medical City, Riyadh, Saudi Arabia; ^4^ Department of Internal Medicine, King Abdulaziz Hospital, MNGHA, King Abdullah International Medical Research Center (KAIMRC), Al-Ahsa, Saudi Arabia; ^5^ Department of Electrophysiology, King Abdulaziz Cardiac Center, King Abdullah International Medical Research Center (KAIMRC), MNGHA, King Abdulaziz Medical City, Riyadh, Saudi Arabia

**Keywords:** ventricular tachcardia, ventricular fibrilation, ventricular ablation, mitral valve prolaps, premature ventricle contraction/complex, late enhancement gadolinium

## Abstract

**Background:**

Mitral valve prolapse (MVP) is a common condition, typically benign, but in a small subset of patients, it may lead to life-threatening arrhythmias and sudden cardiac death (SCD). This arrhythmogenic MVP phenotype is often associated with bileaflet prolapse, mitral annular disjunction (MAD), and myocardial fibrosis identified via late gadolinium enhancement (LGE) on cardiac MRI.

**Case Summary:**

Our patient is a 49-year-old man presented with monomorphic ventricular tachycardia and near-syncope. Echocardiography showed bileaflet MVP, MAD and mild mitral regurgitation. Cardiac MRI revealed fibrosis in the papillary muscle. Electrophysiological study (EPS) confirmed inducible ventricular fibrillation (VF) triggered by papillary muscle PVCs. Catheter ablation was successfully performed, eliminating the arrhythmic focus. Despite successful ablation, an implantable cardioverter-defibrillator (ICD) was implanted for secondary prevention, given the high-risk structural substrate. The patient remained arrhythmia-free over 2 years of follow-up.

**Discussion:**

This case highlights critical diagnostic markers—bileaflet prolapse and LGE—associated with arrhythmogenic MVP. While ablation may suppress triggers, it does not completely eliminate the underlying substrate. Current expert consensus supports ICD implantation in patients with sustained VT/VF or sudden cardiac arrest, regardless of ablation success. Management should be individualized based on risk profile, imaging findings, and clinical presentation.

**Conclusion:**

Malignant MVP warrants comprehensive evaluation with echocardiography, cardiac MRI, and EPS. Catheter ablation is effective in eliminating arrhythmic foci, but ICD therapy remains essential for secondary prevention. Future high-quality trials and clear guidelines for diagnosis, risk stratification, and management are essential to avoid both over- and under-treatment, ensuring optimal outcomes for the patients with MVP.

## Introduction

Mitral valve prolapse (MVP) is a common valvular abnormality, typically considered benign. However, a subset of patients have arrhythmogenic or “malignant” MVP, in which MVP is associated with complex ventricular arrhythmias and even sudden cardiac death (SCD) despite often minimal hemodynamic impairment (Basso et al., 2015). The annual incidence of sudden death in MVP is low (estimated <1% per year), but MVP has been identified at autopsy in up to 4%–7% of unexplained sudden deaths in young people (Sabbag et al., 2023). Recent research has focused on identifying high-risk MVP patients and understanding the mechanisms linking MVP to ventricular arrhythmias. Key structural factors–such as bileaflet prolapse, mitral annular disjunction (MAD), and ventricular fibrosis–have emerged as important risk markers (Pistelli et al., 2023). Advanced imaging modalities and electrophysiological studies now play a central role in evaluating these patients. Here we present a case with bileaflet MVP, MAD, and papillary muscle fibrosis who manifested with monomorphic ventricular tachycardia (VT). The case highlights how integration of multimodality imaging and an electrophysiology (EP) study was used to pinpoint the arrhythmia source, enabling successful catheter ablation of triggering PVCs from the papillary muscle. We discuss this patient’s management in the context of the current literature, emphasizing the mechanistic links between MVP anatomy and arrhythmias, and reviewing emerging approaches to risk stratification and therapy (including advanced mapping-guided ablation).

## Case presentation

A 49-year-old man with no significant past medical history presented with recurrent palpitations and an episode of near-syncope. He was physically active and had no known family history of cardiac disease or sudden death.

### Initial evaluation

**FIGURE 1:**
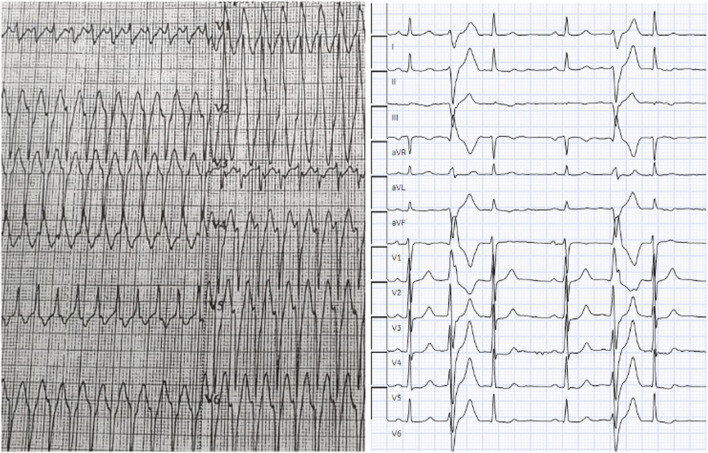
Wide Complex Tachycardia and Premature Ventricular Contractions on Electrocardiogram (ECG). The initial ECG shows a wide complex tachycardia during the patient’s presentation to emergency department. Subsequent ECGs showed complex premature ventricular contractions (PVCs) with right bundle branch block (RBBB) morphology and superior axis. Abbreviations: ECG, electrocardiogram; PVC, premature ventricular contraction; MVP, mitral valve prolapse; VT, ventricular tachycardia; SCD, sudden cardiac death.

On presentation to our emergency department, his blood pressure was 90/55 mmHg. Cardiac auscultation revealed a prominent mid-systolic click followed by a late-systolic murmur at the apex, consistent with mitral valve prolapse. The initial 12-lead electrocardiogram showed a wide complex tachycardia, which was managed with direct current cardioversion, resulting in conversion to sinus rhythm with frequent premature ventricular contractions (PVCs). A follow-up ECG demonstrated frequent PVCs with a right bundle branch block morphology and superior axis, without evidence of pre-excitation or QT prolongation (Figure 1).

### Diagnostic work-up

**FIGURE 2:**
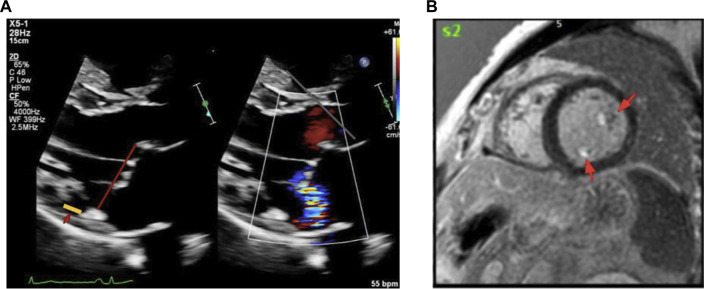
Transthoracic echocardiography (TTE) and Cardiac MRI Showing Fibrosis in Mitral Valve Prolapse. **(A)** shows the parasternal long-axis view during end-systole shows bileaflet mitral valve prolapse with MAD, highlighted by the yellow line. **(B)** shows cardiac MRI demonstrates late gadolinium enhancement in the papillary muscles and mitral annulus, indicating areas of fibrosis in a patient with malignant mitral valve prolapse. Abbreviations: MAD, mitral annular disjunction; MRI, magnetic resonance imaging.

Given the concern for malignant arrhythmia, the patient underwent transthoracic echocardiography demonstrating bileaflet MVP with MAD, and mild mitral regurgitation. The prolapse was more pronounced in the posterior leaflet–the hinge point of the posterior leaflet was visibly displaced away from the left ventricular myocardium in the end systole. Left ventricular size and systolic function were within normal ranges (ejection fraction >55%), with no regional wall motion abnormalities. Then a cardiac magnetic resonance (CMR) study was performed. CMR confirmed the bileaflet prolapse, mild regurgitation and a patchy fibrosis in the posterior papillary muscle, regions corresponding to the mitral apparatus. There were no other fibrosis foci or infiltrative patterns on CMR (Figure 2).

### Electrophysiological study and ablation

The patient was referred for an electrophysiology study and possible ablation in light of his high-risk features. During EP testing frequent PVCs were observed originating from the LV posteromedial papillary muscle (consistent QRS morphology with RBBB pattern and superior axis). Programmed stimulation with isoproterenol infusion reproducibly induced a rapid polymorphic ventricular tachycardia (VT) degenerating to ventricular fibrillation (VF) consistent with an arrhythmogenic substrate which then was successfully cardioverted. This confirmed that the papillary muscle PVCs were not only causing monomorphic VT but also serving as triggers for VF in this patient.

**FIGURE 3:**
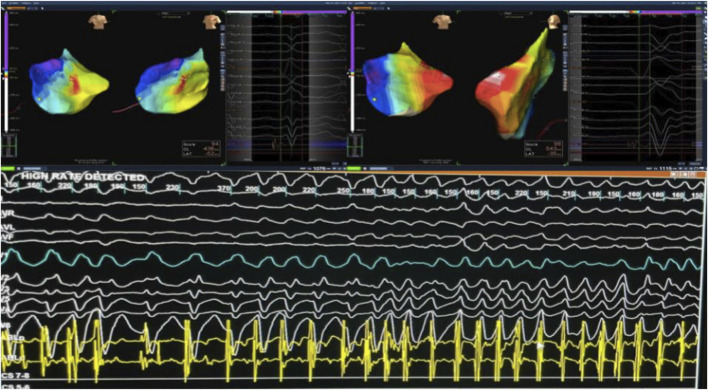
Targeting Arrhythmogenic Zones During VT mapping in The Posterior Mitral Valve Papillary Muscle. During mapping of PVCs from the posterior mitral valve papillary muscle, VT was induced and degenerated into VF, which was successfully cardioverted. Abbreviations: PVC, premature ventricular contraction; VT, ventricular tachycardia; VF, ventricular fibrillation.

Electroanatomical mapping of the LV was performed using a 3D mapping system with a contact force-sensing catheter. Transesophageal echocardiography (TEE) was utilized to visualize the papillary muscles in real time and to facilitate precise catheter positioning on the highly mobile papillary structure. Earliest activation of the clinical PVC was mapped to the mid-region of the posteromedial papillary muscle. Ablation at this site was guided by TEE to maintain stable contact on the papillary muscle and by contact force feedback to ensure adequate lesion formation. Radiofrequency lesions (with up to 40 W, irrigated catheter) were delivered, titrated to eliminate the PVCs. During ablation, the PVC frequency gradually reduced and the focus was rendered electrically inactive; no VT or VF could be induced thereafter with aggressive stimulation. The procedure achieved acute success, with complete abolition of the triggering papillary muscle ectopy and non-inducibility of any sustained arrhythmia (Figure 3).

### Implantable cardioverter defibrillator (AICD) decision

Given the presence of multiple high-risk features including bileaflet mitral valve prolapse with MAD, myocardial fibrosis, complex ventricular ectopy with inducible ventricular fibrillation during electrophysiological study, and a clinical history of near-syncope—a detailed discussion was held with the patient regarding the risk of arrhythmic recurrence and the potential benefits of implantable cardioverter-defibrillator (ICD) therapy for secondary prevention of sudden cardiac death. Following shared decision-making, the patient opted to proceed with ICD implantation, which was performed successfully without complications.

### Follow-up

Over the 2 years following catheter ablation and ICD implantation, the patient experienced significant symptomatic improvement. Device interrogation at 6-month intervals revealed no episodes of sustained arrhythmia or delivered shocks. Continued surveillance with device interrogation and follow-up echocardiography was scheduled as part of the long-term management plan.

## Discussion

### Arrhythmogenic mechanisms and risk factors in MVP

MVP has been linked to ventricular arrhythmias through a combination of mechanical, structural, and electrical factors. The prolapsing redundant leaflets and papillary muscles can impose abnormal mechanical stretch on the underlying myocardium, especially in systole. Over time, this is thought to lead to low-grade inflammation and fibrotic changes in the LV myocardium, particularly in the basal inferior wall and papillary muscle regions​. Histopathology from young sudden death victims with MVP has consistently shown fibrosis of the papillary muscles and inferobasal LV wall in these patients. In a landmark autopsy series by Basso et al., 100% of arrhythmic MVP SCD cases had papillary muscle fibrosis and 88% had inferobasal fibrosis; notably, 70% had bileaflet involvement of the mitral valve (Basso et al., 2015). Fibrosis is a key arrhythmogenic substrate, as it can disrupt myocardial electrical conduction and promote reentrant circuits or act as a focus for ventricular ectopy. Indeed, the presence of LGE on cardiac MRI (reflecting replacement fibrosis) is now recognized as a powerful risk marker. A recent meta-analysis of 12 studies (1,464 patients) found that MVP patients with LGE had nearly a 3-fold higher risk of ventricular arrhythmias compared to those without fibrosis (Tang and Fan, 2024). Another important element in arrhythmogenic MVP is Mitral Annular Disjunction (MAD) – an abnormal separation between the mitral annulus and LV myocardium. MAD alters the mechanical dynamics of the mitral apparatus; during systole, the mitral annulus can exhibit an exaggerated posterior motion or “curling.” This motion is believed to exacerbate stress on the papillary muscles and adjacent myocardium. MAD is frequently seen in arrhythmic MVP patients and is considered part of the malignant MVP phenotype. However, MAD by itself is common in MVP and can even be seen in some normal individuals, so its independent prognostic value is still under study (Sabbag et al., 2023). It likely acts in concert with other factors (like leaflet redundancy and fibrosis) to increase arrhythmic risk.

Patients with bileaflet MVP (as opposed to single leaflet prolapse) are overrepresented in cohorts with complex arrhythmias. The classic malignant MVP patient profile was first described by Sriram et al. as predominantly female, middle-aged, with bileaflet myxomatous prolapse, frequent ventricular ectopy, and characteristic ECG repolarization changes. In their series of otherwise idiopathic cardiac arrest survivors, 42% had MVP; those with bileaflet prolapse were 90% female and often showed inferior T wave inversions and complex ventricular ectopy (Sriram et al., 2013). Bileaflet (Barlow’s type) MVP often features thicker, redundant leaflets and greater annular abnormalities, which may predispose to more pronounced mechanical stretch and fibrosis. It is noteworthy, however, that not all studies find a female predominance; for example, a large 595-patient MVP cohort by Essayagh et al. found arrhythmic events were actually more common in men, despite MVP being more common in women (Essayagh et al., 2020). This suggests there may be two phenotypes of arrhythmogenic MVP: one younger, female, without severe regurgitation (Barlow’s disease) and another older, often male, with fibroelastic degeneration and significant regurgitation Enriquez Sarano et al., 2024. Both phenotypes share common risk markers such as bileaflet involvement, MAD, and fibrosis.

Electrocardiographic markers can offer important clues. T-wave inversions (TWI) or biphasic T waves in the inferior (II, III, aVF) and lateral leads are commonly noted in arrhythmic MVP patients (Sriram et al., 2013). Frequent PVCs, couplets, or nonsustained VT on ambulatory monitoring are another red flag. These PVCs often have a polymorphic character, with multiple morphologies suggesting different focal triggers (e.g., one from a papillary muscle, another from the Purkinje network). In Sriram et al.‘s malignant MVP patients, Holter recordings showed frequent bigeminy and runs of ventricular tachycardia; notably, PVCs with alternating origins from the outflow tract and papillary/fascicular regions were present (Sriram et al., 2013). Purkinje fiber involvement is thought to be an additional mechanism–areas of fibrosis in MVP may irritate the Purkinje network, and Purkinje-triggered PVCs can initiate polymorphic VT/VF (Jin et al., 2024).

In summary, arrhythmogenic MVP (often termed “MVP complex”) is characterized by a constellation of risk factors. Key identified risk markers include.

•Bileaflet mitral valve prolapse (redundant, myxomatous leaflets involving both anterior and posterior leaflets) (Sriram et al., 2013).
•Mitral annular disjunction (MAD) with systolic annular *curling* motion (Sabbag et al., 2023).
•Evidence of fibrosis on cardiac MRI (late gadolinium enhancement in papillary muscles or inferolateral LV) (Tang and Fan, 2024).
•ECG repolarization abnormalities (inferolateral T-wave inversions or ST segment depression) (Essayagh et al., 2020).
•Complex ventricular ectopy (multifocal PVCs, NSVT) on Holter monitoring (Sriram et al., 2013).

It is important to note that no single factor perfectly predicts risk, and even the presence of multiple features does not guarantee malignant events. However, patients who harbor several of the above features should be considered for closer monitoring and preventative strategies (Pistelli et al., 2023).

### Diagnostic evaluation of arrhythmic MVP

Given the subtle structural changes and often normal global cardiac function in MVP patients, high-yield diagnostic techniques are needed for risk stratification.

#### Electrocardiography and ambulatory monitoring

A 12-lead ECG in arrhythmic MVP may be normal or show nonspecific ST-T changes. However, as noted, T-wave inversions in the inferior and lateral leads are a common finding in high-risk patients (reflecting underlying fibrosis or regional repolarization disturbance). Patients with arrhythmogenic MVP often exhibit characteristic premature ventricular contractions (PVCs) on ECG. These typically have a RBBB morphology, reflecting their origin in the left ventricle, and a characteristic frontal-plane axis that helps localize the ectopic focus. In MVP-related PVCs, the axis is usually inferior or superior depending on whether the focus is more anterior or posterior in the left ventricle. In general, PVCs arising from the anterolateral papillary muscle or anterior mitral annulus tend to have an inferior or rightward axis (positive QRS in leads II, III, aVF). In contrast, PVCs from the posteromedial papillary muscle or posterior/inferior mitral annular region display a superior (leftward) axis (negative in leads II and III) (Sabbag et al., 2023). Long-term ambulatory ECG (Holter) monitoring is essential to quantify ventricular ectopy. Detection of complex ventricular arrhythmias (couplets, polymorphic PVCs, or runs of VT) in an MVP patient should prompt further evaluation (Sriram et al., 2013). Some researchers have even explored using artificial intelligence on ECGs to identify MVP patients at arrhythmic risk, although this remains investigational (Tison et al., 2023). In our case, the patient’s presenting ECG revealed sustained VT, corroborating the presence of an active arrhythmogenic substrate.

#### Echocardiography

Transthoracic echocardiography (TTE) is the cornerstone for MVP diagnosis and can also provide important risk information. Standard 2D echocardiography will confirm prolapse (systolic billowing of leaflets >2 mm beyond the annular plane) and assess mitral regurgitation severity. For risk stratification, echocardiographers look for leaflet redundancy/thickening (Barlow’s disease) as opposed to thin leaflets, since the former is associated with more arrhythmic events (Esposito et al., 2024). The presence of mitral annular disjunction can be identified on echocardiography, typically in long-axis views as a systolic separation between the hinge point of the posterior leaflet and the ventricular myocardium. Care must be taken to distinguish true MAD from the artifact of the leaflet tissue apposing the atrial wall (Esposito et al., 2024). In our patient, bileaflet mitral valve prolapse (MVP) consistent with Barlow’s disease was observed, characterized by thickened, redundant leaflets and mild to moderate mitral regurgitation. The posterior leaflet exhibited more prominent prolapse, with systolic displacement of its hinge point away from the left ventricular myocardium. Echocardiography can also reveal secondary signs of arrhythmic burden, such as subtle left ventricular dysfunction or dilation that might result from chronic PVCs or tachycardia.

Advanced echo techniques have further enhanced risk assessment.

•Speckle-Tracking Strain Echocardiography: Strain imaging can unmask regional mechanical abnormalities not seen on gross inspection. Arrhythmic MVP patients often have increased mechanical dispersion, meaning different LV segments contract out of sync. An early clue is post-systolic shortening in the basal inferolateral segment (continued contraction after aortic valve closure), indicating delayed regional contraction due to fibrosis or stretch. A pronounced disparity in timing of peak strain between segments has been associated with ventricular arrhythmias. Speckle-tracking can quantify this; one study noted that MVP patients with high arrhythmic burden had greater standard deviation of time-to-peak strain (dispersion) than those without arrhythmias (Esposito et al., 2024).
•Tissue Doppler Imaging (TDI): The Pickelhaube sign is a striking TDI finding named after the spiked German helmet. It is defined as a spiked, high-velocity late-systolic signal (generally ≥16 cm/s) at the lateral mitral annulus on pulsed-wave TDI. This reflects abrupt systolic contraction of the papillary muscle pulling on the annulus. Muthukumar et al. (who first described this sign) and others found it to be a specific marker of malignant MVP, correlating with frequent ventricular ectopy (Esposito et al., 2024; Muthukumar et al., 2017).
•Three-Dimensional Echocardiography: 3D echo provides *en face* visualization of the mitral valve and annulus throughout the cardiac cycle. It can accurately measure the degree of leaflet billow and annular dynamics. Studies using 3D TTE have quantified MAD length and found that a greater MAD (especially >8–10 mm) often correlates with ventricular arrhythmias. 3D echo can also identify leaflet scallop prolapse more precisely and any systolic curling of the ventricular wall. Essayagh et al. in 2018 demonstrated that 3D echo could detect MAD in the majority of degenerative MVP and that MAD was linked with more pronounced systolic annular motion abnormalities (Sabbag et al., 2023). While the clinical significance of MAD’s exact length is still debated, 3D echo is a valuable tool for comprehensive MVP assessment (Essayagh et al., 2020).

#### Cardiac magnetic resonance (CMR)

CMR has become indispensable for evaluating arrhythmic MVP (Esposito et al., 2024). Late gadolinium enhancement sequences can directly visualize myocardial fibrosis. In arrhythmogenic MVP, CMR frequently shows LGE in a characteristic distribution: the inferior basal LV wall (just underlying the posterior leaflet) and often extending into or around the papillary muscles (Basso et al., 2015). Even diffuse interstitial fibrosis (not evident as discrete LGE) can be detected by T1 mapping and extracellular volume (ECV) quantification. CMR is superior to echocardiography for detecting and measuring MAD, especially if the disjunction is small. In one recent prospective study, Nagata et al. (2022) reported that 95% of MVP patients with complex arrhythmias had LGE on CMR, versus a much lower proportion in those without arrhythmias (Nagata et al., 2022). The extent of scar was often substantial; of those with LGE, 85% had fibrosis in the LV myocardium and about 50% had papillary muscle enhancement (Esposito et al., 2024). In our patient, CMR confirmed the fibrosis that was suspected from echo and ECG cues. Importantly, CMR also helps exclude alternative arrhythmic substrates–in this case we ruled out arrhythmogenic right ventricular cardiomyopathy (ARVC), myocarditis, or other cardiomyopathies that can cause ventricular arrhythmias. Current recommendations are to obtain a CMR in any MVP patient with unexplained ventricular arrhythmia or high-risk features, as finding fibrosis (even if LVEF is normal) changes the risk stratification (Sabbag et al., 2023). So in summary arrhythmogenic MVP, CMR is essential for detecting myocardial fibrosis, with late gadolinium enhancement (LGE) observed in 95% of patients with complex arrhythmias. Among those with LGE, 85% show fibrosis in the left ventricular myocardium and approximately 50% exhibit papillary muscle enhancement. LGE most commonly appears in the basal inferolateral LV wall and papillary muscles. CMR also outperforms echocardiography in identifying mitral annular disjunction (MAD), particularly when subtle. These findings underscore the importance of CMR in risk stratification, especially in MVP patients with unexplained ventricular arrhythmias or high-risk features, even when ejection fraction is preserved.

#### Electrophysiological studies (EPS)

An invasive EPS can clarify the arrhythmic mechanism in MVP and guide therapy. If a patient has sustained VT or survived VF (as in our case), an EPS is often performed to map PVC triggers or inducible arrhythmias that might be amenable to ablation. One hallmark of arrhythmogenic MVP is that the PVCs triggering VF often originate from the papillary muscles or nearby Purkinje network. Enriquez et al. studied 25 patients with arrhythmic MVP and papillary muscle PVCs: they found Purkinje potentials in all patients and identified papillary muscle foci as VF triggers in those with cardiac arrest (Enriquez et al., 2019). In their series, nearly half of the patients who underwent CMR had LGE in the papillary muscle, highlighting the correspondence between scar and the electrophysiologic substrate (Jin et al., 2024). An EPS can also test inducibility of VT/VF. While inducibility is not a perfect predictor of spontaneous arrhythmias, if sustained polymorphic VT or VF is easily inducible in an MVP patient, it underscores a high-risk substrate.

#### Electroanatomic mapping and noninvasive mapping

During EPS, 3D electroanatomic mapping helps locate the origins of PVCs. Papillary muscle arrhythmias present a challenge due to the complex geometry and motion of the papillary muscles. Specialized techniques, such as intracardiac echocardiography (ICE) guidance, are often employed to ensure the ablation catheter maintains contact on the papillary muscle tip (Mahmoodi et al., 2024). The use of contact force-sensing catheters and even cryoablation has been advocated to improve lesion delivery on these moving targets (Sabbag et al., 2023). Noninvasive mapping modalities, like body surface ECG mapping (ECG imaging), have been explored in research settings to localize PVC exit sites in MVP without an invasive procedure (Tison et al., 2023). These technologies can construct activation maps of the ectopic beats from multi-lead ECG recordings and may identify a papillary muscle or fascicular source. While promising, noninvasive mapping is not yet routine clinical practice for MVP; invasive EPS remains the gold standard when an intervention (ablation) is being considered.

In our case, the EPS confirmed the suspicions from noninvasive tests: a papillary muscle PVC was the culprit initiating VF, and successfully ablating that focus abolished the inducible arrhythmia. This highlights how invasive mapping can not only diagnose the mechanism but also provide therapy in the same setting.

### Management and risk stratification

Management of arrhythmogenic MVP is challenging because it requires balancing the low absolute event rate against the potentially catastrophic outcome of SCD in a subset of patients​ (Sabbag et al., 2023). There are no randomized trials specific to malignant MVP, so current management is guided by observational studies and expert consensus​ (Sabbag et al., 2023; Mahmoodi et al., 2024). A comprehensive approach includes lifestyle advice, medical therapy for arrhythmia suppression, catheter ablation in select cases, and consideration of ICD for prevention of SCD. In patients with severe valvular regurgitation, timely mitral valve surgery is indicated per standard guidelines, and there is interest in whether correcting the MVP might secondarily reduce arrhythmias.

#### Medical therapy

Symptom control and modest suppression of premature ventricular contractions (PVCs) can be achieved with beta-blockers or verapamil (Ling et al., 2014). For patients requiring a more substantial reduction in PVC burden, class Ic and III antiarrhythmic drugs—such as flecainide, propafenone, and amiodarone—have shown greater efficacy, often accompanied by improvements in left ventricular function (Stec et al., 2012). However, long-term use of amiodarone carries a considerable risk of toxicity, necessitating cautious, individualized use. While sotalol may also reduce PVC frequency, its impact on LV function has been limited (Anderson et al., 1986).

#### Catheter ablation

Catheter ablation has emerged as a valuable therapy in arrhythmic MVP, especially for reducing PVC burden and eliminating triggers for malignant arrhythmias. The goal is typically to ablate the PVC foci that are suspected to trigger VF or cause intolerable ventricular ectopy. As in our case, successful ablation of a papillary muscle PVC can prevent further VF episodes. Several studies have shown that ablation is feasible in this setting, though with some caveats. Papillary muscle VT/PVC ablation is technically challenging–success rates in MVP range from about 60% to 84% in various series (Sabbag et al., 2023). In a study by Enriquez et al. (2019) on 25 patients, acute success was achieved in the majority, but they noted the need for meticulous technique (often using ICE guidance) due to the complex anatomy (Enriquez et al., 2019). Another series by Bumgarner et al. at the Cleveland Clinic reviewed 43 MVP patients who underwent ablation and/or ICD; ablation was acutely successful in 65% of cases, but 26% had a recurrence of ventricular tachycardia or fibrillation during 2.5-year follow-up (Bumgarner et al., 2019). Recurrences often arose from a different location than the initial target, underscoring the possibility of a progressive arrhythmogenic cardiomyopathy in MVP whereby new arrhythmic foci can emerge over time (Mahmoodi et al., 2024). Thus, while ablation can markedly improve arrhythmia control and perhaps delay or prevent ICD therapies, patients require continued monitoring afterward. In summary catheter ablation in arrhythmogenic MVP has demonstrated acute success rates ranging from 60% to 84%, with long-term arrhythmia-free survival reported in 65% of patients over a 2.5-year follow-up. However, recurrence of ventricular tachycardia or fibrillation occurred in 26% of cases, often due to the emergence of new arrhythmic foci, reflecting the progressive nature of the underlying cardiomyopathy. While ablation can significantly reduce arrhythmic burden and potentially delay or avoid ICD therapies, continued long-term monitoring remains essential.

Indications for ablation in MVP are individualized. Current expert consensus suggests that PVC/VT ablation is reasonable in patients with frequent symptomatic PVCs or non-sustained VT that is refractory to medical therapy or not tolerated. It is also advised in cases of PVC-induced cardiomyopathy (where reducing PVC burden can recover LV function) and in those who suffer arrhythmias despite an ICD (to reduce ICD shocks) (Sabbag et al., 2023). In our patient, ablation was indicated given the clear culprit PVC initiating VF and the patient’s age (hoping to avoid a lifetime of recurrent ICD shocks). Notably, ablation in MVP should be done in centers experienced with papillary muscle arrhythmias and in close coordination with imaging specialists–sometimes intra-procedural echo or MRI integration is used to target scar regions.

#### Implantable cardioverter-defibrillator (ICD)

The role of ICDs in arrhythmogenic MVP centers on prevention of sudden death. ICDs are unequivocally indicated for secondary prevention–any MVP patient who has survived a cardiac arrest or sustained VT with hemodynamic compromise should receive an ICD, as per standard guidelines (Sabbag et al., 2023). Our patient met criteria for secondary prevention and was appropriately offered the option of (ICD) placement. The challenging question is primary prevention, should an ICD be implanted in an MVP patient who has never had sustained VT/VF, but who is deemed high-risk by other markers? There are no specific guidelines for primary prevention ICD in MVP outside of the usual indications (like LVEF ≤35%, which typically does not apply here since most have preserved EF) (Mahmoodi et al., 2024). The 2022 EHRA consensus recommends that MVP patients should not be considered as having a “benign” idiopathic arrhythmia if they have significant risk features–in other words, the presence of fibrosis (LGE on CMR) or frequent ventricular arrhythmias means the substrate is not truly normal (Sabbag et al., 2023). In the absence of randomized data, decisions must be individualized. The consensus statement suggests an ICD should be strongly considered for an MVP patient with unexplained syncope plus frequent complex ventricular ectopy or nonsustained VT, especially if additional risk factors (like LGE on MRI or MAD with bileaflet flail) are present. For example, an otherwise healthy MVP patient with bileaflet prolapse, MAD, inferolateral LGE, and runs of NSVT might be offered an ICD before a catastrophic event occurs–essentially treating them analogously to other cardiomyopathies with high SCD risk (Sabbag et al., 2023). This is a case-by-case decision, weighing the ICD’s risks and impact on quality of life against the estimated SCD risk.

#### ICD after catheter ablation

If a patient undergoes successful ablation of PVCs/VT, is an ICD still necessary? This remains a grey area. Ablation does not negate the underlying disease process; as noted, new arrhythmogenic areas can develop. Therefore, most experts err on the side of caution. In patients who already meet secondary prevention criteria (resuscitated arrest or sustained VT), an ICD is indicated regardless of ablation success (Sabbag et al., 2023). In those who have not had a sentinel event but undergo ablation for frequent PVCs or NSVT, the decision is harder. Some centers would still favor ICD implantation if the patient had very high-risk features (say extensive fibrosis on MRI or prior syncope), whereas others might opt for close follow-up without ICD, given the ablation has addressed the known triggers. The Cleveland Clinic series (Bumgarner et al.) illustrated that many patients managed with ablation alone did well, but a subset had recurrent VT that would have benefited from an ICD shock had it occurred (Bumgarner et al., 2019). In our case, despite successful ablation and the absence of inducible arrhythmias post-procedure, an ICD was implanted owing to the patient’s initial presentation with sustained ventricular tachycardia, high-risk structural features on imaging—including bileaflet prolapse and myocardial fibrosis—and the patient’s informed decision favoring secondary prevention of sudden cardiac death. Generally, post-ablation ICD strategies should be individualized: if a patient had life-threatening arrhythmia, ICD is usually kept or placed; if the ablation was purely for symptomatic PVCs in a patient with no other risk, one might defer ICD and monitor.

### Mitral valve surgery

For patients with MVP and severe mitral regurgitation, prompt surgical repair or replacement is indicated per standard valve guidelines, irrespective of arrhythmia. Interestingly, there have been observations that after mitral valve repair, some patients have a reduction in ventricular ectopy and fewer ICD shocks. The relief of prolapse and annular dilation might reduce the mechanical stretch on the LV. A small series even reported doing surgical cryoablation of papillary muscle arrhythmia foci during mitral valve surgery, with encouraging acute results (Mahmoodi et al., 2024). However, surgery solely to mitigate arrhythmia (in the absence of significant regurgitation) is not established. It remains an area of investigation whether repairing the valve in a high-risk MVP patient (even if regurgitation is moderate) could be a prophylactic strategy to prevent arrhythmias. Until more data emerge, the primary role of surgery is to treat mechanical dysfunction (MR), with any arrhythmic benefit being a bonus. Our patient had only mild MR, so surgery was not indicated; his management focused on arrhythmia control.

### Follow-up and outlook

Arrhythmogenic MVP requires long-term follow-up with a multidisciplinary approach. Patients should be periodically reassessed with ECG/Holter (to check for recurrence or new arrhythmias) and imaging if there is a change in clinical status. The progressive nature of myocardial changes in MVP means that a patient with negative LGE 1 year could develop fibrosis later–though significant changes likely span years. There’s interest in serial MRI or advanced echo for very high-risk patients, but no consensus on interval. ICD checks (if implanted) and symptom surveillance are mainstays. Patients are also educated about CPR and family screening: while MVP is often sporadic, there are familial cases, and first-degree relatives may be evaluated for MVP and low-risk ECG features, though malignant MVP itself is not strongly hereditary in most instances (Pistelli et al., 2023).

#### Ongoing research

The growing clinical recognition of arrhythmogenic MVP has spurred major NIH-funded research initiatives aimed at improving early detection and risk stratification. The Icahn School of Medicine at Mount Sinai received a $10.2 million NHLBI R01 grant (R01HL166720) in September 2023 to investigate PET/MRI-based imaging and myocardial fibrosis markers for early identification of high-risk MVP (Icahn School of Medicine at Mount Sinai, 2023).

## Conclusion

Arrhythmogenic MVP is a unique cause of malignant ventricular arrhythmias, characterized by a mechanistic link between valvular prolapse and myocardial fibrosis. This case highlights several key lessons. First, meticulous imaging and electrical evaluation can identify high-risk MVP patients before a catastrophic event–in our patient, features like MAD, papillary muscle fibrosis, and VF on EPS signposted the malignant potential. Second, papillary muscle PVCs can act as triggers for life-threatening arrhythmias in MVP, and advanced EP mapping is capable of localizing and eliminating these triggers. The use of TEE or intracardiac echo is vital for successful ablation on a moving papillary muscle, aligning with recent improvements reported in the literature. Third, therapy should be multifaceted: catheter ablation can substantially reduce arrhythmia burden and ICD shocks in arrhythmic MVP, but an ICD for secondary prevention remains essential given the possibility of recurrence or new foci. Finally, an integrated approach to discussion of the literature is crucial–rather than viewing this as an isolated case, we illustrate how the patient’s presentation and management reflect the broader spectrum of arrhythmogenic MVP syndrome as described in recent studies. Ongoing research and collaboration (as called for in recent consensus statements) will hopefully refine the risk stratification and management of MVP patients. Until then, cases like this underscore the importance of recognizing high-risk MVP features and the value of early, aggressive intervention (including consideration of EP study and ablation) to prevent SCD. This case, therefore, adds to the growing body of evidence that arrhythmia in MVP can be effectively targeted when guided by precise imaging and electrophysiologic mapping, ultimately improving patient outcomes.

## Data Availability

The original contributions presented in the study are included in the article/supplementary material, further inquiries can be directed to the corresponding author.
